# Remediating Soil Lead with Fish Bones

**DOI:** 10.1289/ehp.120-a20a

**Published:** 2012-01-01

**Authors:** Kris S. Freeman

**Affiliations:** Kris S. Freeman has written for *Encarta* encyclopedia, NIH, ABCNews.com, and the National Park Service. Her research on the credibility of online health information appeared in the June 2009 *IEEE Transactions on Professional Communication*.

Fish bones are made of the phosphate mineral apatite, which readily combines with lead to form pyromorphite, a stable crystalline mineral that can’t be absorbed by the human digestive system.^1^^,^^2^ Now researchers are using fish bones and other phosphate-rich amendments to remediate lead in urban soils. “We have seen reduction in bioaccessibility in some lab samples up to fifty percent within just a few weeks of treatment,” says Steve Calanog of the U.S. Environmental Protection Agency (EPA), who is overseeing an agency project using fish bones to clean up soils in the South Prescott neighborhood of Oakland, California.^3^

*In situ* and laboratory tests have shown that phosphates can also immobilize other potentially toxic metals, including copper, zinc, cadmium, and uranium.^1^^,^^4^^,^^5^^,^^6^^,^^7^^,^^8^ In one of these studies, lead concentrations in soil leachate treated with fish bones dropped from 0.28 mg/L to 0.00065 mg/L within weeks.^6^^,^^7^ Unlike phosphate fertilizers, the apatite in fish bones does not run off the soil.^2^ Fish bones also are being used as a phosphate source for lead remediation projects in other urban areas, including a pilot project in New Orleans, Louisiana, funded by the U.S. Department of Housing and Urban Development.^9^

Excessive blood lead can cause delays in neurological and physical development in children, and high blood pressure, kidney problems, and cancer in adults. Lead added to house paints and fuels decades ago still lingers in urban neighborhoods across the country, and children can be exposed through the soil in yards and playgrounds. Total lead levels in soils tested in the Oakland/San Francisco Bay area average 300–600 ppm, according to Calanog. The U.S. EPA considers lead in bare soil in play areas to be a hazard at a concentration of 400 ppm,^10^ and the California Office of Environmental Health Hazard Assessment has estimated that exposure to a soil lead concentration of 80 ppm will produce an increase in blood lead of up to 1 μg/dL.^11^ In South Prescott, which is adjacent to the AMCO Chemical Superfund site, total soil lead levels average 843 ppm, with some spots reaching 2,500 ppm.^3^

The 2-year, $4-million South Prescott project is part of a growing trend to treat soils contaminated with lead and other heavy metals in place, rather than removing and replacing soils or capping them with asphalt or concrete, techniques that require dump sites to store contaminated soils as well as sources of uncontaminated soils for replacement. “Lead contamination is a pervasive problem, and our traditional ways of responding are neither economically nor ecologically sustainable,” Calanog says.

Phosphate immobilization is not recommended for sites with lead levels above 4,000 ppm, such as those heavily contaminated with lead paint, according to Rufus Chaney, a research agronomist with the U.S. Department of Agriculture Environmental Management and Byproduct Utilization Laboratory. Chaney has worked on soil remediation projects in South Prescott, Baltimore, and other urban neighborhoods, and on the development of less expensive tests for measuring levels of bioaccessible lead.^12^

In South Prescott, workers till 3 pounds of fish bones into each square foot of property treated, then cover the freshly tilled soil with 3–6 inches of clean soil and plants.^3^ Many project workers are hired locally through the Cypress Mandela Training Center, a pre-apprenticeship program in West Oakland. Workers receive health and safety and lead-abatement training, and they wear personal protective equipment, including respirators, Calanog says.

The fish bones used in South Prescott come from commercial processing of pollock into fillets, fish sticks, and artificial crab meat. Catfish farms are another possible source of fish bones for remediation, according to Judith Wright, the geologist whose studies of fossilized fish bones^13^ inspired her invention (U.S. Patent #6217775) of the process of using fish bones to remediate heavy metals. Wright, owner of PIMS NW, Inc., supplies the cleaned fish bones for projects such as that at South Prescott. Bones from weight-bearing animals, such as cattle, contain the same chemical form of apatite as do fish bones but in a more highly crystallized form that combines less readily with metals, Wright says.

“Many amendments other than fish bones can also be used for phosphate immobilization,” Chaney says. Indeed, the EPA, U.S. Army Corps of Engineers, U.S. Department of Defense, and other agencies have tested numerous synthetic, mined, and organic sources of phosphates for remediation, including mineral apatite, rock phosphate, soluble phosphate fertilizers, and biosolids compost from treated sewage.^1^^,^^4^^,^^5^^,^^6^^,^^7^^,^^8^ Calanog says many of these possible amendments are fairly accessible in terms of cost, but the beauty of the fish bones, according to Wright, is that they’re free of contaminants. Using fish bones also avoids ecological issues involved with mining phosphates, and the bones will not dissolve but “will remain in place to stabilize metals for millennia,” she says.^2^

Chaney favors the use of organic amendments such as composts that are high in iron, as well as phosphates. Whereas phosphates transform lead into other compounds, iron compounds physically bind with lead through adsorption, a process in which one compound adheres to the surface of another. When lead molecules adsorb to iron-based compounds such as ferrihydrite, he says, they are no longer soluble and can’t be absorbed through the lining of the small intestine. Without realizing it, many urban gardeners are already remediating their soils by adding fertilizers and composts that contain phosphates and iron. “Only about five to ten percent of lead in urban gardens is bioavailable, compared to fifty to sixty percent in urban soils elsewhere,”^12^ Chaney says.
